# Efficacy of Biologics in Aspirin Exacerbated Respiratory Disease

**DOI:** 10.1002/clt2.70212

**Published:** 2026-10-03

**Authors:** Jennifer K. Priessnitz, Anastasia Hagopian, Alison J. Patev, Joseph K. Han, Kent K. Lam

**Affiliations:** ^1^ Division of Internal Medicine Macon & Joan Brock Virginia Health Sciences at Old Dominion University Norfolk Virginia USA; ^2^ Department of Medicine Eastern Virginia Medical School at Old Dominion University Norfolk Virginia USA; ^3^ Research and Infrastructure Service Enterprise Macon & Joan Brock Virginia Health Sciences at Old Dominion University Norfolk Virginia USA; ^4^ Division of Allergy Macon & Joan Brock Virginia Health Sciences at Old Dominion University Norfolk Virginia USA; ^5^ Division of Division of Rhinology & Endoscopic Sinus and Skull Base Surgery Macon & Joan Brock Virginia Health Sciences at Old Dominion University Norfolk Virginia USA

**Keywords:** aspirin‐exacerbated respiratory disease, biologic therapy, biologics, narrative review, type 2 inflammation

## Abstract

**Background:**

Aspirin‐exacerbated respiratory disease (AERD), also termed nonsteroidal anti‐inflammatory drug exacerbated respiratory disease (NERD), is characterized by asthma, chronic rhinosinusitis with nasal polyposis, and hypersensitivity to cyclooxygenase‐1 inhibitors, frequently resulting in severe, treatment‐refractory airway disease. Biologic therapies targeting type 2 inflammation are increasingly used in AERD; though their relative efficacy remains incompletely defined.

**Methods:**

We conducted a semi‐systematic narrative review of biologic therapies in AERD, guided by established narrative review frameworks. PubMed/Medline, ScienceDirect, and ClinicalTrials.gov were searched for studies reporting clinical outcomes in AERD/NERD or clearly defined AERD/NERD subgroups. This review synthesizes current evidence and presents an evidence‐informed clinical framework to support biologic selection in patients with AERD.

**Results:**

Eighteen studies identified, including randomized controlled trials, observational studies, and real‐world studies. Across these studies, dupilumab was most consistently associated with improvements in both upper and lower airway outcomes. Anti–IL‐5 and anti–IL‐5Rα therapies reduced eosinophilia and asthma exacerbations, with variable sinonasal benefit. Omalizumab demonstrated efficacy in selected atopic phenotypes and in conjunction with aspirin therapy following desensitization. Emerging subgroup data suggests that Tezepelumab may reduce asthma exacerbations in AERD.

**Conclusions:**

Current evidence suggests biologic therapies provide meaningful benefit in AERD, although available evidence is limited by small sample sizes and heterogeneous designs. Prospective comparative studies are needed to inform optimal biologic selection and sequencing.

**Abbreviations**
15‐HETE: 15‐hydroxyeicosatetraenoic acid
15‐LOX‐1: 15‐lipoxygenase‐1
15‐oxo‐ETE: 15‐oxo‐eicosatetraenoic acid
ACQ: Asthma Control Questionnaire
ACT: Asthma Control Test
AERD: aspirin‐exacerbated respiratory disease
ATAD: aspirin therapy after desensitization
COX‐1: cyclooxygenase‐1
CRSwNP: chronic rhinosinusitis with nasal polyps
ED: emergency department
FcεRI: high‐affinity IgE receptor
FDA: Food and Drug Administration
FeNO: fractional exhaled nitric oxide
FEV_1_
: forced expiratory volume in one second
IgE: immunoglobulin E
IL‐13: interleukin‐13
IL‐4: interleukin‐4
IL‐5: interleukin‐5
IL‐5Rα: interleukin‐5 receptor alpha
ILC2: group 2 innate lymphoid cells
LMS: Lund–Mackay score
LTE_4_
: leukotriene E_4_
NCS: nasal congestion score
NERD: nonsteroidal anti‐inflammatory drug–exacerbated respiratory disease
NPS: nasal polyp score
NSAID: nonsteroidal anti‐inflammatory drug
OCS: oral corticosteroids
PGE_2_
: prostaglandin E_2_
QoL: quality of life
RCT: randomized controlled trial
SABA: short‐acting beta agonist
SNOT‐22: 22‐item Sino‐Nasal Outcome Test
T2: type 2
TSLP: thymic stromal lymphopoietin
UPSIT: University of Pennsylvania Smell Identification Test

## 1 Introduction

Aspirin exacerbated respiratory disease (AERD), or nonsteroidal anti‐inflammatory drug (NSAID)‐exacerbated respiratory disease (NERD), is defined by the triad of asthma, chronic rhinosinusitis with nasal polyps (CRSwNP), and respiratory hypersensitivity reactions to aspirin and NSAIDs [1, 2]. Although AERD and NERD are often used interchangeably, AERD will be used throughout this review except when referring to studies that use the term NERD. AERD affects approximately 7% of adults with asthma, with a prevalence of up to 14% in severe asthma, and typically presents in early to mid‐adulthood with severe, steroid‐dependent asthma and recurrent sinonasal disease [1, 3, 4, 5]. Asthma in patients with AERD, like aspirin‐tolerant asthma, may demonstrate either eosinophilic or non‐eosinophilic airway inflammation when classified using induced sputum [6, 7]. However, patients with severe AERD more commonly exhibit systemic type 2 inflammatory features, including peripheral blood eosinophilia, elevated type 2 (T2) cytokine activity through increased IL‐4, IL‐5, and IL‐13 signaling, proving a rationale for targeting T2 pathways with biological therapies [8]. Recent mechanistic studies further demonstrate that epithelial‐derived cytokines, mast cell activation, and dysregulated eicosanoid signaling collectively sustain the inflammatory cascade characteristic of AERD, reinforcing the therapeutic rationale for biologic agents targeting type 2 immune pathways [9, 10].

Although biologic therapy has become an increasingly important component of AERD management, current recommendations are largely extrapolated from chronic rhinosinusitis with nasal polyps and severe asthma guidelines, including the European Position Paper on Rhinosinusitis and Nasal Polyps (EPOS 2020) [11], EAACI position statements [12], AAAAI guidance documents [13, 14, 15], and GINA recommendations [16]. This narrative review synthesizes these guideline recommendations alongside available evidence from randomized controlled trials, observational cohorts, and real‐world studies evaluating biologic efficacy in AERD. We present a practical evidence informed framework that integrates the available literature to assist clinicians in selecting biologic therapy based on patient characteristics and disease manifestations. In addition to comparing the efficacy and safety of currently available biologics, this review examines how clinical phenotype, inflammatory biomarkers, and disease manifestations may inform individualized biologic selection. We further discuss emerging precision medicine approaches and propose an evidence‐informed clinical framework for phenotype‐based treatment selection and monitoring in AERD.

### 1.1 Pathophysiology of AERD

AERD is characterized by dysregulation of arachidonic acid metabolism, resulting in excess cysteinyl leukotriene production and reduced prostaglandin E_2_ (PGE_2_) signaling [1]. Protective PGE_2_ signaling is diminished due to cyclooxygenase‐1 (COX‐1) inhibition and intrinsic defects in prostaglandin synthesis, leading to mast cell and eosinophil over‐activation contributing to excessive T‐2 inflammation and acute respiratory reactions [17]. This imbalance promotes airway inflammation through enhanced leukotriene‐mediated bronchoconstriction and recruitment of eosinophils and mast cells to the respiratory mucosa [9, 10, 18]. In addition to altered prostaglandin metabolism, increased expression of leukotriene receptors and elevated urinary leukotriene E4 levels have been observed in AERD patients, further supporting the central role of leukotriene‐driven inflammation in disease pathogenesis [9, 19]. Therapies such as aspirin treatment and dupilumab have been shown to partially restore PGE_2_ signaling, underscoring the clinical relevance of this pathway [20, 21]. These findings highlight how targeted biologic therapies may interrupt upstream inflammatory signaling pathways and modify both upper and lower airway manifestations of the disease [10, 22].

Recent investigations have further expanded the understanding of arachidonic acid dysregulation in AERD by implicating the 15‐lipoxygenase‐1 (15‐LOX‐1) pathway [10]. Airway epithelial cells highly express 15‐LOX‐1, which converts arachidonic acid to 15‐hydroxyeicosatetraenoic acid (15‐HETE). Through epithelial–mast cell transmetabolism, 15‐HETE is subsequently converted to 15‐oxo‐eicosatetraenoic acid (15‐oxo‐ETE), a bioactive lipid mediator increasingly recognized as a contributor to the dysregulated eicosanoid network characteristic of AERD [10, 23]. Although the precise biologic function of 15‐oxo‐ETE remains under investigation, this pathway further illustrates the complexity of lipid mediator imbalance beyond excess cysteinyl leukotriene production [10, 23, 24, 25].

In addition to eosinophils and mast cells, emerging evidence has identified innate immune cells, including macrophages and group 2 innate lymphoid cells (ILC2s), as important contributors to AERD pathogenesis. Airway epithelial‐derived alarmins, particularly IL‐33, IL‐25, and thymic stromal lymphopoietin (TSLP), activate ILC2s, resulting in rapid secretion of IL‐5 and IL‐13, eosinophilic recruitment, mucus hypersecretion, and amplification of type 2 inflammation [19]. Macrophages further contribute by interacting with epithelial cells and other innate immune populations to sustain chronic airway inflammation and tissue remodeling [25]. These interconnected pathways provide additional mechanistic rationale for biologic therapies targeting IL‐4/IL‐13, IL‐5, and TSLP signaling in patients with AERD.

### 1.2 Purpose

While previous narrative reviews [26] have explored the efficacy of biologics for the treatment of AERD, these reviews have not provided a comparison of effectiveness and potential adverse effects across various biologics. The current narrative review uses a comparative effectiveness framework to compare the overall effectiveness, benefits, and potential adverse effects of each individual biologic, and create a summary useful for clinicians when considering biologic use for the treatment of AERD. By synthesizing evidence across randomized trials, observational cohorts, and real‐world studies, this review aims to provide a clinically practical overview of therapeutic outcomes relevant to both upper and lower airway disease manifestations.

## 2 Methods

We performed a semi‐systematic narrative review of biologic therapies for AERD. A narrative review approach was selected due to the heterogeneity of study designs, limited AERD‐specific subgroup analyses, and the author hopes to pursue a formal meta‐analysis in the future. PubMed/Medline, ScienceDirect, and ClinicalTrials.gov were searched for peer‐reviewed studies using combinations of terms of “aspirin‐exacerbated respiratory disease,” “AERD,” “NSAID‐exacerbated respiratory disease,” “NSAID‐ERD,” “NERD,” “Samter's Triad,” “aspirin sensitive asthma,” “biologics,” “biologic therapy,” “omalizumab,” “mepolizumab,” “benralizumab,” “dupilumab,” “tezepelumab” and “efficacy.” Studies from January 2015 to January 2026 were prioritized. This time frame was selected to capture contemporary clinical evidence corresponding to the emergence and expanded use of biologic therapies in asthma and CRSwNP management [27].

Eligible studies were required to be available in English and report clinical efficacy outcomes of biologic therapies in patients with AERD or clearly defined AERD subgroups. Randomized controlled trials, observational studies, case series, and relevant clinical trial data were included. Studies were excluded if they did not address AERD, lacked measurable efficacy outcomes, or did not include an identifiable AERD population. When multiple publications reported outcomes from the same patient cohort, the most comprehensive or recent analysis was included to avoid duplication of data.

Data extraction was performed by one reviewer, with independent verification of study inclusion and key outcomes by a second reviewer. This dual‐review process was implemented to improve accuracy in study selection and minimize potential bias during data extraction [28]. Data were synthesized qualitatively. Outcome measures evaluated at least one of the following: nasal polyp score (NPS), nasal congestion score (NCS), Sino‐Nasal Outcome Test (SNOT‐22), University of Pennsylvania Smell Identification Test (UPSIT), Lund‐Mackay (LMS), forced expiratory volume in 1 second (FEV_1_), Asthma Control Questionnaire (ACQ), number of exacerbations requiring Emergency Department (ED) visit or hospitalization, need for surgery and/or systemic steroids, and assessing the efficacy on aspirin/NSAID challenge reactivity, where reported. These outcome measures were selected because they represent validated clinical endpoints commonly used to assess disease burden and treatment response in both asthma and CRSwNP populations [11]. Inclusion and exclusion criteria are summarized in a PRISMA‐style flow chart in Figure 1, and characteristics of included studies (*n* = 18) are listed in Table 1. The treatment framework presented in this review was developed by the authors through synthesis of the published literature and existing clinical recommendations and is intended to illustrate practical application of current evidence rather than establish new clinical practice guidelines.

**FIGURE 1:**
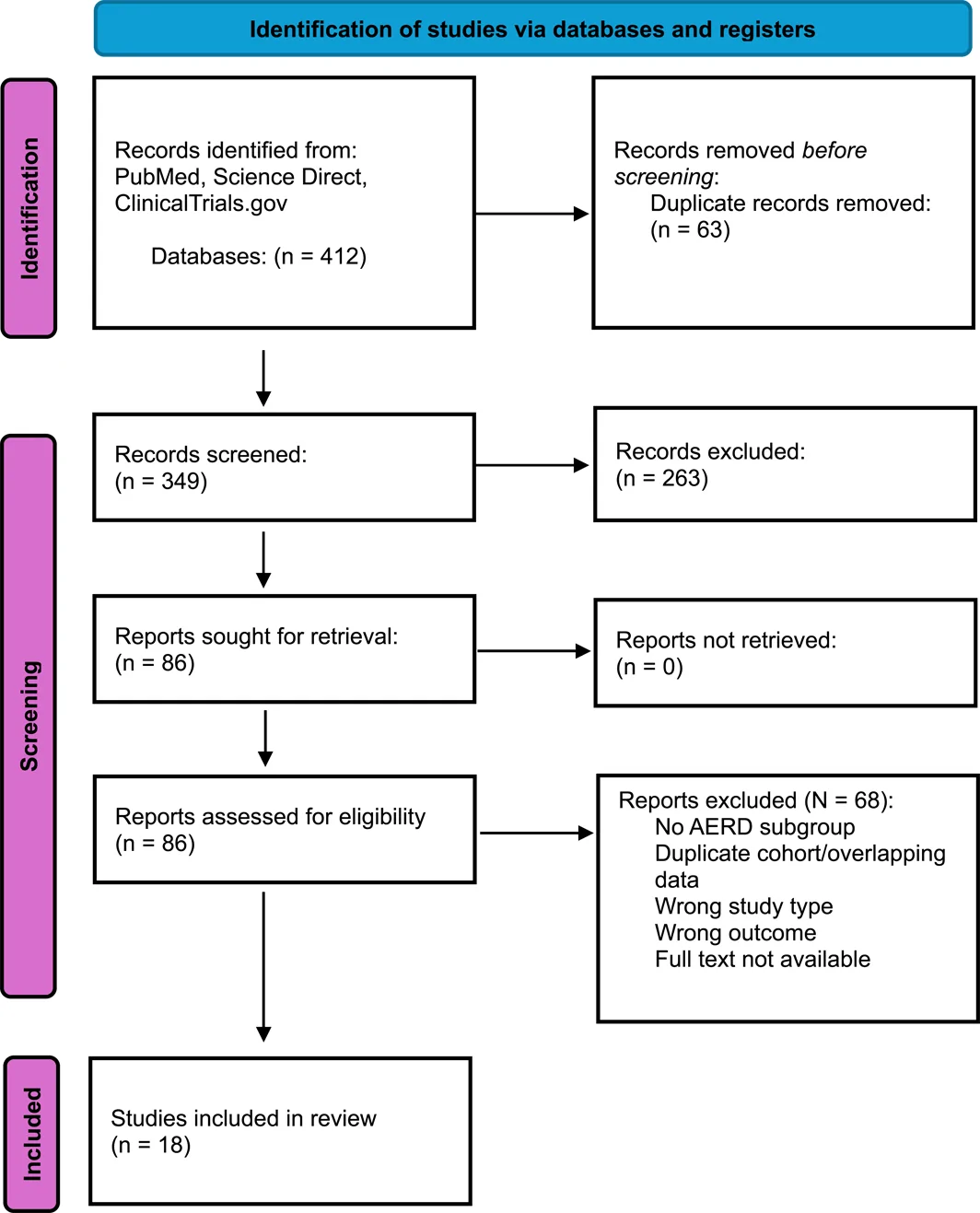
PRISMA‐informed table showing the identification of eligible studies and reasons for exclusion in the narrative review.

**TABLE 1:** Biologics in AERD: literature review table.

Authors (Year)	Study design	Biologic(s)	Sample description	Key outcomes (AERD‐relevant)
Hayashi et al. (2020) [17]	Randomized, double‐blind, placebo‐controlled crossover trial	Omalizumab	*n* = 16 adults with AERD; aspirin therapy after desensitization (ATAD) on drug versus placebo	Increased aspirin tolerance (62.5% tolerated 930 mg cumulative on omalizumab vs. 0% placebo); decreased urinary LTE_4_ during challenge; fewer respiratory symptoms.
Jean et al. (2019) [29]	Retrospective outcomes study	Omalizumab	*n* = 29 AERD; 1‐year outcomes after initiation	86% had fewer oral corticosteroids (OCS) or short acting beta agonist (SABA) use; 62% fewer OCS (median 2 → 0); FEV_1_ stable to slightly increased; greatest benefits in atopic AERD.
Gevaert et al. (2020) [30]	Two phase III RCTs (CRSwNP) with AERD subgroup POLYP1/2	Omalizumab	Adults with CRSwNP (AERD subgroup *n* = 72/265, 27.6%); 2 year outcomes in comparison to placebo	Decreased NPS and daily NCS; improved SNOT‐22 and UPSIT versus placebo; supports sinonasal benefit including in AERD subgroup.
Damask et al. (2022) [31]	Subgroup analysis of phase III randomized control trial (RCT) POLYP1/2	Omalizumab	CRSwNP trial participants; subgroups including AERD *n* = 72/265 (27.5%); 2 year outcomes in comparison to placebo	Confirmed efficacy on NPS, NCS, SNOT‐22, UPSIT in relevant subgroups including AERD.
Buchheit et al. (2022) [20]	Prospective cohort (mechanistic + clinical)	Dupilumab	*n* = 22 adults with AERD	Large decrease in SNOT‐22 (34 points at 3 months); UPSIT improved (73% regained normal/near‐normal smell); nasal LTE_4_ decreased; nasal PGE_2_ increased; FEV_1_ ↑ (+12.6% predicted at 4 weeks). ACQ +1.3.
Bachert et al. (2019) [32]	Two phase III RCTs SINUS‐24 and SINUS‐52; (CRSwNP) with AERD subset	Dupilumab	Adults with severe CRSwNP; included AERD patients	Reduced NPS and sinus opacification; improved smell and symptoms; consistent benefits across subgroups (supports AERD efficacy).
Mustafa & vadamalai (2021) [33]	Case series (pre/post)	Dupilumab	*n* = 5 AERD with repeat aspirin challenge after ≥ 3 months therapy	Higher aspirin dose tolerance on dupilumab versus baseline.
Achanta et al. (2025) [34]	Retrospective cohort	Dupilumab	AERD with persistent symptoms despite successful ATAD	Addition of dupilumab improved SNOT‐22 (−21), ACT (+4.3), reduced OCS use (−61%)
Bavaro et al. (2021) [35]	Real‐world cohort (switch study)	Dupilumab after prior anti‐IL‐5/IL‐5R failure	AERD with inadequate response to mepolizumab/reslizumab/benralizumab	Improved SNOT‐22 and ACT; reduced annualized exacerbations after switch to dupilumab.
Tuttle et al. (2016, 2018) [3, 36]	Retrospective cohort	Mepolizumab	*n* = 14 AERD at tertiary center; ≥ 3 doses mepolizumab	SNOT‐22 decreased (17.7 points); improved congestion/anosmia; ACT +5.1; trend toward FEV_1_ increase +5‐6%; fewer exacerbations; allowed OCS tapering; no sinus surgeries during follow‐up.
Han et al. (2021) [37]	Phase III RCT (CRSwNP)	Mepolizumab	CRSwNP adults; ∼27% AERD (∼108/407)	Reduced endoscopic polyp score; 57% lower risk of sinus surgery versus placebo
Carr et al. (2024) [3, 36]	Phase III RCT (severe asthma) NAVIGATOR subgroup analysis	Tezepelumab (anti‐TSLP)	Severe asthma; AERD subgroup analyzed	83% reduction in annualized exacerbation rate in AERD subgroup versus placebo; benefits across eosinophil strata.
Yamashita et al. (2024) [39]	Single‐patient case report	Tezepelumab (after benralizumab)	AERD with persistent CRSwNP/anosmia despite benralizumab	Marked improvement in congestion and smell; reduction in nasal polyps, normalization of FeNO and eosinophils after switch to tezepelumab.
Corren et al. (2023) [40]	RCT pooled analysis of PATHWAY and NAVIGATOR	Tezepelumab	Asthma with AERD subgroup	Improved FEV_1_ by ∼0.23 L more than placebo in the overall asthma population, and this improvement was consistent in those with AERD. Better asthma control scores and quality of life, with no specific data.
Wangberg et al. (2022) [41]	Multicenter real‐world comparative cohort	Omalizumab, mepolizumab, reslizumab, benralizumab, dupilumab	*n* = 74 AERD on biologics	Only dupilumab showed significant improvements (SNOT‐22, ACT, exacerbations); high discontinuation with anti‐IL‐5/IL‐5R and omalizumab; 98% remained on dupilumab long‐term.
Kumar et al. (2026) [42]	Multicenter real‐world utilization study	Omalizumab, dupilumab, mepolizumab, benralizumab,	AERD patients	Higher discontinuation with ATAD than biologics (−40% vs. −20%), 25% transitioned from ATAD to biologic for inadequate control of symptoms or intolerance
Cihanbeylerden et al. (2025) [43]	Retrospective comparative cohort	Omalizumab, mepolizumab, benralizumab	70 AERD patients on ATAD	ATAD + biologic improvements versus ATAD: ACT (+5.0), SNOT‐22 (−14 points), OCS reduction −38%
Wu et al. (2022) [44]	Network meta‐analysis (CRSwNP)	Dupilumab versus omalizumab versus mepolizumab	CRSwNP RCTs pooled; AERD proportion variable	Largest effect on NPS with dupilumab (−2.0) versus omalizumab (−1.1) versus mepolizumab (−0.9); supports dupilumab for aggressive polyp disease.

*Note:* A summary of RCTs, real‐world cohorts, case series, and meta‐analyses evaluating omalizumab, dupilumab, mepolizumab, reslizumab, benralizumab, and tezepelumab for AERD patients with key AERD‐relevant outcomes (SNOT‐22, UPSIT, NPS/NCS, FEV_1_, exacerbations, OCS use, and aspirin tolerance). Of note, studies lacking AERD specified patients were excluded from this table.

Abbreviations: AERD, aspirin‐exacerbated respiratory disease; ATAD, aspirin therapy after desensitization; CRSwNP, chronic rhinosinusitis with nasal polyps; ED, emergency department; FEV_1_, forced expiratory volume in one second; IgE, immunoglobulin E; IL, interleukin; LMS, Lund–Mackay score; LTE_4_, leukotriene E_4_; NCS, nasal congestion score; NPS, nasal polyp score; OCS, oral corticosteroids; RCT, randomized controlled trial; SABA, short‐acting beta agonist; SNOT‐22, 22‐item Sino‐Nasal Outcome Test; UPSIT, University of Pennsylvania Smell Identification Test.

## 3 Results

The following sections summarize the clinical efficacy of individual biologic therapies to contextualize the current evidence known for AERD treatment. Because no international guideline is dedicated exclusively to AERD, current management recommendations are derived from guidelines addressing CRSwNP and severe asthma. EPOS 2020 recommends biologic therapy for patients with severe uncontrolled CRSwNP meeting established eligibility criteria, while GINA recommends biologics according to severe asthma phenotype [11, 16]. EAACI and AAAAI guidance similarly recognize biologics as important steroid‐sparing therapies in appropriately selected patients with type 2 inflammation [12, 13, 14]. Although these documents include patients with AERD, biologic selection is generally individualized because comparative evidence remains limited.

### 3.1 Omalizumab (Anti‐IgE)

#### 3.1.1 Pharmacodynamics

Omalizumab is a humanized monoclonal antibody that binds circulating IgE, preventing high‐affinity IgE receptor (FcεRI) mediated mast cell and basophil activation [45]. By reducing the availability of free IgE, omalizumab also downregulates FcεRI receptor expression on effector cells, thereby decreasing cellular sensitivity to allergen‐mediated activation [46]. In AERD, IgE blockade may reduce mast cell reactivity and attenuate aspirin‐induced mediator release [17]. This mechanism is particularly relevant in AERD because mast cell activation contributes to leukotriene release and broncho constrictive responses following exposure to cyclooxygenase‐1 inhibitors [9, 19].

#### 3.1.2 Upper Airway Symptoms

Although omalizumab is approved for chronic rhinosinusitis with nasal polyposis (CRSwNP), AERD‐specific sinonasal outcomes remain limited. In the POLYP1 and POLYP2 Phase 3 trials, omalizumab demonstrated significant improvements in daily NCS and NPS over 24 weeks. NPS scores improved 1.14 and 0.59 points in POLYP1 and POLYP2 respectively, and NCS scores improved by 0.55 and 0.5 points in POLYP1 and POLYP2 trials. Improvements were also observed in SNOT‐22 (−16.02 and −15.04) and olfactory function measured by UPSIT (+3.81 and + 3.86), and total nasal symptoms improved by 1.91 points between the two trials [4, 30, 31]. Post hoc analysis of POLYP1 and POLYP2 demonstrated improvement in sinonasal symptoms and better sense of smell in some patients on omalizumab, but results were variable [47]. These findings suggest that while IgE blockade can improve sinonasal inflammation in many patients with CRSwNP, responses among individuals with AERD may be heterogeneous due to the complex inflammatory mechanisms underlying the disease [9, 19]. Notably, many AERD patients receiving omalizumab continued to require sinus surgeries or aspirin desensitization, implying incomplete control of upper airway disease with IgE blockade alone [48].

#### 3.1.3 Lower Airway Symptoms

Clinical evidence suggests that omalizumab can improve asthma control and possibly mitigate NSAID reactions in AERD. In a retrospective study of 29 AERD patients, 86% had a reduction in either oral corticosteroids (OCS) or rescue short‐acting beta agonist (SABA) inhaler use one year after starting omalizumab [29]. Specifically, 62% had fewer OCS, dropping from a median of 2 to 0. Benefits were most pronounced in AERD patients with coexistent IgE mediated allergic asthma. Lung function, FEV_1_, tended to be stable or slightly improved under omalizumab, though small sample sizes limit conclusions [29]. These observations are consistent with omalizumab's established efficacy in allergic asthma populations, where IgE neutralization reduces exacerbation frequency and improves symptom control [49].

#### 3.1.4 Aspirin Therapy After Desensitization

Omalizumab also has been shown to attenuate aspirin therapy after desensitization (ATAD) and NSAID hypersensitivity in AERD. In a randomized controlled crossover trial with 16 AERD patients, 62.5% tolerated a cumulative 930‐mg aspirin challenge after three months of omalizumab, compared with 0% on placebo, accompanied by reduced leukotriene E_4_ (LTE4) levels and fewer respiratory reactions [17]. These findings support the hypothesis that IgE‐mediated mast cell activation contributes to aspirin‐induced reactions and that suppression of IgE signaling may increase aspirin tolerance in selected patients [19].

#### 3.1.5 Adverse Effects

Omalizumab is generally well tolerated; anaphylaxis remains a rare but recognized risk at approximately 0.1%–0.2% [50]. The most commonly reported adverse effects include injection‐site reactions, headache, and mild viral respiratory infections, with rates generally comparable to placebo in clinical trials [51]. Omalizumab's safety profile in asthma and CRSwNP has been well established, and no increase in severe adverse events has been observed in AERD cohorts [29].

#### 3.1.6 Conclusions

In summary, omalizumab tends to reduce asthma exacerbations and OCS requirements in AERD, especially in IgE—mediated or atopic individuals [29]. Its effects on sinonasal symptoms are positive but moderate in comparison to that of newer biologics.

### 3.2 Dupilumab (Anti–IL‐4Rα)

#### 3.2.1 Pharmacodynamics

Dupilumab is a fully human monoclonal antibody targeting the IL‐4 receptor *α* subunit, thereby inhibiting IL‐4 and IL‐13 signaling [52]. Because IL‐4Rα is shared by both cytokine signaling pathways, dupilumab suppresses multiple downstream components of type‐2 inflammation including IgE synthesis, eosinophilic recruitment, and mucus hypersecretion [53]. In AERD, IL‐4/IL‐13 blockage is associated with significant reductions in nasal and urinary LTE_4_ levels and restoration of anti‐inflammatory PGE_2_ signaling, and reduce mucus hypersecretion, epithelial barrier leakiness, and alternative macrophage activation [20, 54]. These effects target central inflammatory mechanisms implicated in AERD pathogenesis [55].

#### 3.2.2 Upper Airway Symptoms

Dupilumab produces rapid improvements within months in CRS among AERD patients. In a prospective post hoc analysis of 22 AERD patients, dupilumab led to an improvement on SNOT‐22 scores (+34 points) after 3 months. Additionally, objective smell testing, measured by the UPSIT, showed 73% of dupilumab treated patients regained a normal or near‐normal sense of smell. Anosmia prevalence dropped from 16 out of 22 patients at baseline, to four out of 22 patients after one month. Endoscopic NPS also regressed significantly after one month [20]. These results align with SINUS‐24 and SINUS‐52 Phase III trials, in which the AERD subgroup achieved significant nasal polyp shrinkage and sinus opacification reduction, evidenced by an increase on the LMS (+6.33 points), compared to placebo. Moreover, participants experienced improvements in NPS scores (+1.89 points), NCS scores (+1.17 points), SNOT‐22 (+24.35 points), and UPSIT (+10.17 points) [32]. Overall, dupilumab consistently improves patient‐reported symptoms, olfaction, and endoscopic disease severity in AERD‐associated CRSwNP [55].

#### 3.2.3 Lower Airway Symptoms

Lower airway outcomes also significantly improve with dupilumab. In a prospective post hoc analysis of 22 AERD patients, there was an increase in FEV_1_ (+12.6%) predicted after one month of therapy, which persisted at three months [20]. Additionally, patients reported better asthma control, with reduction in Asthma Control Questionnaire‐6 (ACQ‐6) scores (−1.3 points; baseline score 1.6) at 3 months [20]. Similarly, a multi‐center retrospective study in AERD patients found dupilumab users had fewer asthma exacerbations. OCS use dropped from a median of two uses per year to zero per year on treatment. These improvements occurred regardless of baseline blood eosinophil count, underscoring dupilumab's efficacy across AERD phenotypes, including those who may not be highly eosinophilic [41].

#### 3.2.4 Aspirin Therapy After Desensitization

In a small case series of five patients undergoing ATAD, dupilumab increased aspirin tolerance following desensitization, with all patients tolerating a higher cumulative dose after at least 3 months of therapy compared to their initial aspirin challenge [33]. Among patients with persistent disease despite ATAD, the addition of dupilumab was associated with further clinical benefit such as improved SNOT‐22 (−21 points), ACT (+4.3 points), and reduced OCS use (−61%), supporting its role as escalation therapy following suboptimal ATAD response [34].

#### 3.2.5 Adverse Effects

Dupilumab is generally well tolerated. It is associated with injection‐site reactions, nasopharyngitis, and transient eosinophilia [56]. A subset of patients, particularly those with atopic dermatitis, experience conjunctivitis (15.8% vs. 8.4% in placebo) or keratitis (1.3% vs. 0.2% in placebo), representing a recognized class effect [57, 58]. In CRSwNP and AERD studies, epistaxis, headache, and arthralgia were occasionally observed, but rates were comparable to placebo, and the majority of events were mild [58, 59, 60].

#### 3.2.6 Conclusions

Overall, dupilumab demonstrates the most consistent multidomain efficacy among available biologics and is frequently favored for patients with severe AERD, particularly those with prominent sinonasal disease. Its ability to inhibit IL‐4 and IL‐13 signaling allows broad suppression of type‐2 inflammation affecting both upper and lower airway disease manifestations [19].

### 3.3 Mepolizumab (Anti–IL‐5) and Reslizumab (Anti–IL‐5)

#### 3.3.1 Pharmacodynamics

Mepolizumab and reslizumab are humanized monoclonal antibodies targeting IL‐5, resulting in depletion of circulating and tissue eosinophils [61]. By neutralizing IL‐5 signaling, these agents reduce eosinophil maturation, survival, and recruitment within airway tissues, thereby attenuating eosinophilic inflammation [62]. Given AERD's hallmark eosinophilia in nasal polyps and airways, anti–IL‐5 therapy is hypothesized to alleviate polyp burden and asthma severity, although patient outcomes were subpar.

#### 3.3.2 Upper Airway Symptoms (Reslizumab and Mepolizumab)

There is sparse literature focusing on reslizumab in AERD. Small case series suggest that reslizumab yields outcomes similar to mepolizumab in patients with CRSwNP, such as reduction in eosinophil counts and improvements in asthma control, although did not show evidence of sinonasal benefit, but with no unique advantages in AERD [41]. Overall, available evidence for reslizumab in AERD remains limited and largely extrapolated from studies of severe eosinophilic asthma [63].

Mepolizumab has been more extensively studied. In a retrospective cohort of 14 patients with AERD, there was a reduction in SNOT‐22 scores (−17.7 points) after three to six months of therapy with improvement of nasal congestion and reduction in anosmia severity, indicating significant sinus symptom relief, and zero subjects required sinus surgery on mepolizumab [3]. Similar results were found in the Phase III SYNAPSE trial for CRSwNP, which included around 27% AERD patients (108/407). Mepolizumab achieved significant reductions in endoscopic polyp scores and a 57% lower risk of requiring sinus surgery versus placebo. Although specific AERD subgroup results were not separately published, the overall efficacy “irrespective of baseline NSAID sensitivity” implies AERD patients responded as well as aspirin‐tolerant patients [37].

#### 3.3.3 Lower Airway Symptoms (Mepolizumab)

In a retrospective cohort of 14 patients with AERD, mepolizumab demonstrated a statistically significant improvement asthma control with an ACT increase (+5.1 points) and trended toward higher FEV_1_ (+5–6%) predicted, though not statistically significant in the small sample size. More strikingly, in patients who continued mepolizumab longer‐term, the annual asthma exacerbation rate dropped, and maintenance OCS necessity diminished in five of seven patients [3, 36]. These benefits align with mepolizumab's known efficacy in severe eosinophilic asthma [64].

#### 3.3.4 Adverse Effects (Mepolizumab, Reslizumab)

Mepolizumab and reslizumab have demonstrated similarly benign safety profiles. Serious adverse events remain uncommon across clinical trials and real‐world cohorts [65]. The most frequently reported adverse events include headache, injection‐site discomfort, and back pain, with rare instances of herpes zoster and mild infection risk [65, 66, 67]. Reslizumab, administered intravenously, carries an additional risk of infusion‐related reactions and occasional asymptomatic creatine phosphokinase elevations [66, 68].

#### 3.3.5 Conclusions

Overall, anti–IL‐5 therapy represents a reasonable option for AERD patients with marked eosinophilia or endorsing uncontrolled asthma symptoms, though responses may be less comprehensive across disease domains than with IL‐4/IL‐13 blockade [3, 62, 63, 64].

### 3.4 Benralizumab (Anti–IL‐5Rα)

#### 3.4.1 Pharmacodynamics

Benralizumab is an afucosylated humanized monoclonal antibody targeting the IL‐5 receptor *α* chain, causing rapid and near complete depletion of eosinophils via antibody‐dependent cell‐mediated cytotoxicity. Binding of benralizumab to IL‐5Rα promotes natural killer cell–mediated apoptosis of eosinophils, resulting in near‐complete depletion of circulating and tissue eosinophils [69]. However, clinical experience in AERD is limited.

#### 3.4.2 Upper Airway Symptoms

Sinus outcomes on benralizumab have been mixed. In the OSTRO study evaluating CRSwNP, 29.2% (121/413) of participants had AERD, benralizumab improved NPS and nasal blockage score, with improvements in SNOT‐22 and UPSIT scores, although AERD‐specific subgroup analysis is lacking [70]. Some patients noted reduced nasal congestion and slower polyp recurrence, but others have ongoing sinus symptoms despite complete blood eosinophil suppression [70]. Case reports of AERD patients on benralizumab describe improvement in asthma control, but with persistence of anosmia and nasal obstruction that resolved with switching to tezepelumab. This is possibly due to residual mast cell or leukotriene activity in the upper airway, indicating that IL‐5Rα blockade alone may be insufficient for total disease control in certain cases [39]. In another study, benralizumab's impact on nasal polyposis is variable as it may significantly help in some patients' sinus disease with reduction in SNOT‐22 scores (−8.91 points) over 24 weeks, but others having little to no change [51]. Ongoing evaluation will clarify its role, possibly in combination with other therapies if monotherapy doesn't address all AERD pathways.

#### 3.4.3 Lower Airway Symptoms

Benralizumab effectively controls the asthma component of AERD by reducing eosinophils to nearly zero [71]. Many AERD patients treated with benralizumab achieve improved asthma outcomes, with improvement of ACT scores and FEV_1_ with reduced exacerbations and ability to wean maintenance OCS, similar to other eosinophilic asthma populations [39, 69].

#### 3.4.4 Adverse Effects

Benralizumab is generally well tolerated, with frequently reported symptoms such as headache, pharyngitis, and injection‐site erythema reported at similar rates to placebo [32, 69]. Because benralizumab induces nearly complete eosinophil depletion, theoretical concerns have been raised regarding increased susceptibility to helminthic infections, although this has not been confirmed in clinical practice [72, 73].

### 3.5 Tezepelumab (Anti‐TSLP)

#### 3.5.1 Pharmacodynamics

Tezepelumab is a monoclonal antibody against thymic stromal lymphopoietin (TSLP), an upstream epithelial–derived alarmin that orchestrates multiple inflammatory pathways relevant to AERD. TSLP is released by airway epithelial cells in response to environmental triggers and promotes activation of dendritic cells, type‐2 innate lymphoid cells, and downstream T2 cytokine signaling [74]. By inhibiting TSLP, tezepelumab attenuates downstream T2 cytokine signaling and mast cell activation, offering a broad, phenotype‐agnostic therapeutic approach [38]. Although not yet specifically approved for AERD, its FDA approval for CRSwNP, its strong efficacy in severe asthma and preliminary data in AERD subsets support it as a promising option [74, 75].

#### 3.5.2 Upper Airway Symptoms

In a recent case report, an AERD patient with persistent nasal polyposis and anosmia on benralizumab was switched to tezepelumab, and within months, there were improvement in ACT scores (+3 points; baseline of 22) and FEV_1_ (+4.2%; baseline of 104.8%). Moreover, blood eosinophils dropped significantly from 1782 to less than 300 cells/μL, and a drop in fractional exhaled nitric oxide (FeNO) from 42 to 8 ppb [39]. Although AERD‐specific upper airway analyses were not analyzed, the Phase 3 WAYPOINT trial demonstrated that tezepelumab produced rapid and sustained reductions in nasal polyp burden (−2.10 points) and NCS (−1.09 points), alongside clinically meaningful improvements in smell (−1.03 points), SNOT‐22 (−28.43 points), and radiographic sinus opacification, with a marked reduction in need for surgery by 92% or systemic OCS, which can be relevant to the refractory sinonasal phenotype characteristic of AERD [74, 76].

#### 3.5.3 Lower Airway Symptoms

In the Phase III NAVIGATOR trial for severe asthma, the AERD subgroup had a marked 83% reduction in annualized asthma exacerbation rate on tezepelumab versus placebo, with benefit observed regardless of baseline eosinophil count [38]. In another clinical trial, tezepelumab improved FEV_1_ by 0.23 L compared to placebo in the overall asthma population, and this improvement was consistent in those with AERD. Patients also reported better asthma control scores and quality of life [40].

#### 3.5.4 Adverse Effects

Tezepelumab has exhibited similar safety outcomes to placebo groups in severe asthma and CRSwNP trials, with the most common adverse events being nasopharyngitis, headache, and injection‐site reactions [40, 56]. Because TSLP functions upstream of multiple inflammatory pathways, tezepelumab may offer broader immunologic modulation than therapies targeting single downstream cytokines [74]. No new safety signals have emerged with longer exposure, and no increased risk of serious infections or hypersensitivity has been observed [36].

#### 3.5.5 Conclusions

Overall, tezepelumab represents a promising option for patients with severe or refractory AERD by managing both upper and lower respiratory effects of AERD patients [64]. It may especially help AERD patients who have mixed inflammatory profiles, such as concomitant non‐T2 inflammation. Because TSLP functions upstream of multiple inflammatory pathways, tezepelumab may offer broader immunologic modulation than therapies targeting single downstream cytokines [74]. It stands as an excellent option for severe AERD patients with incomplete response to more targeted biologics or those with contraindications.

### 3.6 Comparative Effectiveness of Biologics in AERD

Comparative effectiveness data for biologic therapies in AERD are limited and derive primarily from retrospective and observational studies. Most available comparative analyses rely on real‐world observational cohorts because randomized head‐to‐head trials between biologic agents in AERD have not yet been performed [26]. AERD is increasingly recognized as a heterogeneous disease rather than a uniform clinical entity. Although all patients exhibit respiratory reactions to COX‐1 inhibitors, the relative severity of chronic rhinosinusitis with nasal polyps (CRSwNP), asthma, eosinophilic inflammation, and allergic sensitization varies considerably. Consequently, biologic selection should incorporate dominant clinical phenotype in addition to overall disease severity (Figure 2).

**FIGURE 2:**
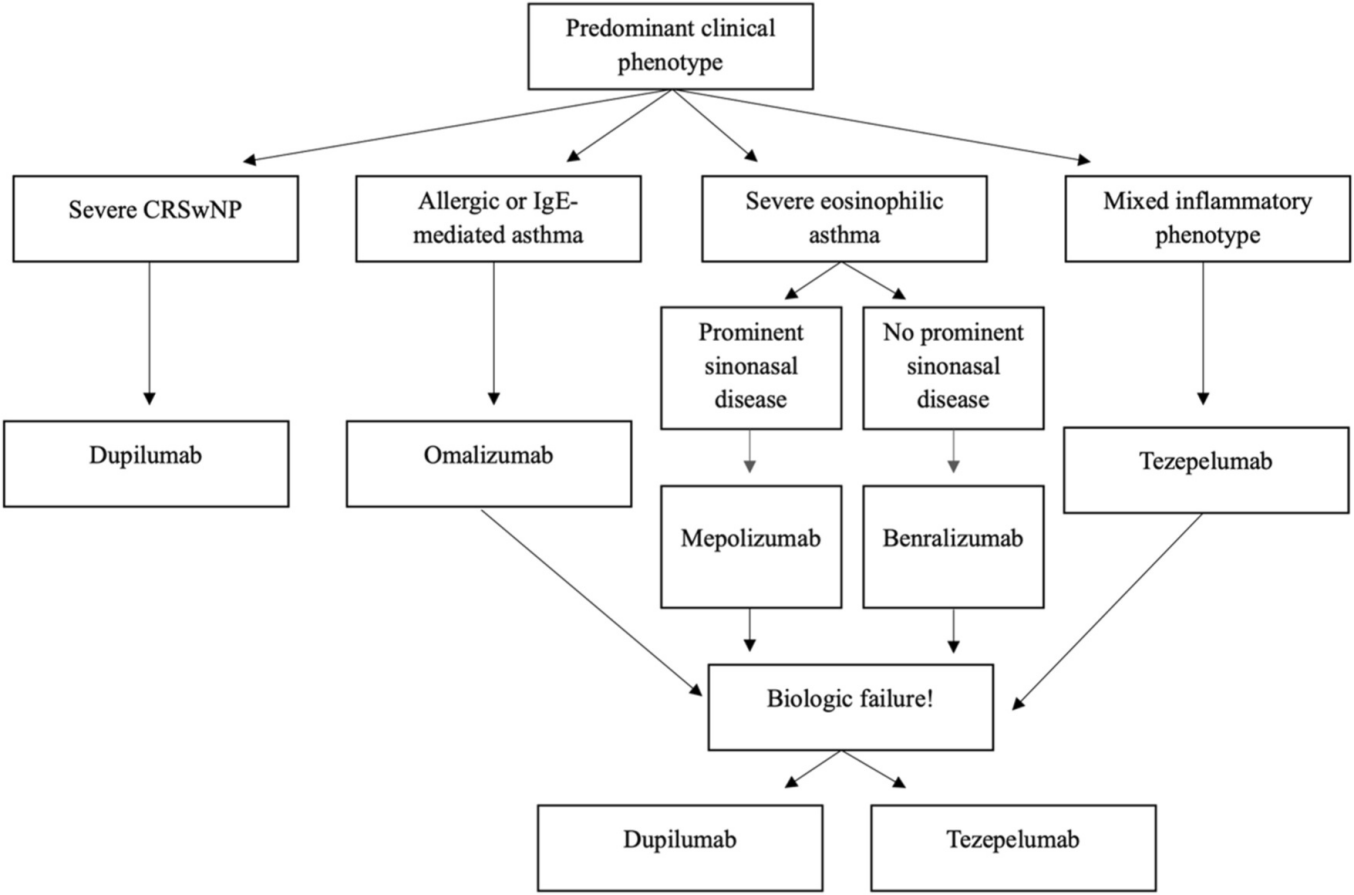
Flow chart outlining biologic efficacy based off AERD phenotype.

In the largest retrospective head‐to‐head comparison to date (*n* = 74), dupilumab was the only biologic associated with significant improvements in sinonasal symptoms and asthma outcomes, whereas anti–IL‐5 and anti‐IgE therapies (omalizumab, mepolizumab, reslizumab, benralizumab) did not demonstrate consistent population‐level benefit [41]. This finding was additionally supported by another study which found AERD patients who failed anti–IL‐5 and anti‐IgE therapies and switched to dupilumab experienced improvements in SNOT‐22, ACT scores, annualized asthma exacerbations [35]. Similarly, in a study of CRSwNP cohorts that included an AERD subgroup, in CRSwNP patients dupilumab produced the largest reduction in NPS score (−2.0 points), followed by omalizumab (−1.1 points) and mepolizumab (−0.9 points) [44]. Network meta‐analyses evaluating biologics for CRSwNP similarly suggest that dupilumab provides the largest improvements in sinonasal outcomes among currently available therapies [77]. Observational data also suggest greater improvement in olfaction and SNOT‐22 with dupilumab compared with omalizumab, although omalizumab appears most effective in selected atopic and allergic asthma AERD phenotypes [41].

In contrast, a subset of patients with predominantly eosinophil‐driven disease achieve durable asthma control on benralizumab monotherapy, highlighting heterogeneity in treatment response and the need for individualized biologic selection [41]. This heterogeneity likely reflects the complex inflammatory pathways involved in AERD, which include both eosinophil‐mediated and mast cell–mediated mechanisms [26, 41]. These differences are reflected in real‐world treatment persistence, with 98% of AERD patients remaining on dupilumab long term, compared with discontinuation rates of approximately 50% for benralizumab and omalizumab and nearly 70% for mepolizumab and reslizumab, largely due to inadequate sinonasal response [41].

Direct comparisons between IL‐5 and IL‐5Rα inhibitors are lacking, with available evidence suggesting broadly similar efficacy and selection often guided by dosing considerations and clinician experience [41]. No head‐to‐head studies have compared tezepelumab with other biologics in AERD; however, in the NAVIGATOR trial, tezepelumab reduced annualized asthma exacerbations by 83% in patients with AERD and demonstrated efficacy across a broad range of inflammatory phenotypes, including those with low eosinophil counts, supporting its potential role in patients with mixed or non‐eosinophilic disease [38, 74].

## 4 Biomarkers

### 4.1 Blood Eosinophils

Blood eosinophil counts remain the most widely available biomarker of type 2 inflammation and may help identify patients most likely to respond to anti‐IL‐5 or anti‐IL‐5 receptor therapies. However, eosinophil counts alone incompletely reflect disease activity in AERD because many inflammatory pathways remain active despite eosinophil depletion [8].

### 4.2 FeNo

FeNO reflects ongoing IL‐4/IL‐13 signaling and may identify patients more likely to benefit from dupilumab or tezepelumab [78, 79, 80].

### 4.3 Urinary LTE_4_

Urinary LTE_4_ is among the most characteristic biomarkers of AERD and reflects dysregulated arachidonic acid metabolism. Elevated urinary LTE_4_ levels correlate with disease activity and aspirin‐induced reactions and may provide additional information regarding inflammatory burden, although routine clinical implementation remains limited because of assay availability and lack of standardized treatment thresholds [81].

### 4.4 Serum IgE

Total serum IgE may support selection of omalizumab in patients with concomitant allergic asthma, although IgE concentration alone should not determine biologic choice [17].

### 4.5 Emerging Biomarkers

In addition to established biomarkers such as blood eosinophils, FeNO, serum IgE, and urinary LTE_4_, several emerging biomarkers may further refine precision medicine approaches in AERD. Increased expression of epithelial‐derived cytokines, including IL‐33 and TSLP, has been implicated in the activation of type 2 inflammatory pathways and ILC2s, making them potential biomarkers of disease activity and therapeutic response [19]. Similarly, periostin has been investigated as a marker of IL‐13–driven airway inflammation, although its clinical utility remains uncertain [82]. More recently, metabolites of the 15‐LOX pathway, including 15‐HETE and 15‐oxo‐ETE, have emerged as promising indicators of dysregulated arachidonic acid metabolism and may improve disease phenotyping [10, 23, 24]. However, these biomarkers remain investigational, and further prospective studies are needed to determine their role in guiding biologic selection and monitoring treatment response.

## 5 Discussion

Biologic therapies have expanded treatment options for AERD, offering meaningful improvements across asthma control, sinonasal disease and patient reported outcomes. Among available agents, dupilumab has demonstrated the most consistent multidomain efficacy, with benefits observed in both upper and lower airway manifestations [26, 41]. The benefits of dupilumab are consistent with findings from the Phase 4 EVEREST clinical trial, wherein patients with severe CRSwNP, 40% of whom also had AERD, where dupilumab plus daily mometasone furoate nasal spray was connected to reduced polyp size and improved smell over 24 weeks [83]. When considering individualization of biologics, patients with prominent CRSwNP and anosmia appear more likely to benefit from IL‐4/IL‐13 blockade [20]. Anti–IL‐5 and anti–IL‐5Rα therapies also improve disease control, primarily through eosinophil depletion, and therefore make them an optimal choice for those with uncontrolled eosinophil‐driven asthma, though sinonasal responses are more variable [3, 41]. Omalizumab remains effective in selected atopic AERD phenotypes by targeting anti‐IgE pathways and may attenuate aspirin‐induced reactions [17], while tezepelumab has emerged as a promising option with broad efficacy across inflammatory profiles [38, 74].

The strength of evidence differs among currently available biologics. Dupilumab has the most extensive evidence in AERD, supported by randomized controlled trials, subgroup analyses, and numerous real‐world studies. Omalizumab and mepolizumab have moderate supporting evidence, including randomized studies and observational cohorts, whereas evidence for benralizumab, reslizumab, and tezepelumab in AERD remains comparatively limited and is largely derived from subgroup analyses, small cohorts, or extrapolation from studies in CRSwNP and severe asthma. Rather than proposing a novel clinical guideline, this review integrates evidence from randomized trials, observational cohorts, and real‐world studies to provide a clinically applicable summary of current biologic therapy for AERD. The accompanying treatment framework is intended to facilitate evidence‐informed clinical decision‐making while recognizing that individualized patient factors and future comparative studies may further refine biologic selection. A comparative summary of available biologics is provided in Table 2.

**TABLE 2:** Outcomes by biologic in AERD/CRSwNP (summary table).

Biologic	FEV1	SNOT‐22	UPSIT	Exacerbations	Steroid use	Polyp size (NPS)	Aspirin tolerance
Omalizumab (anti‐IgE)	↔/↑ small (real‐world) [29]	↓ versus placebo in CRSwNP; AERD subgroup benefited [30, 31]	↑ smell versus placebo (CRSwNP; AERD subgroup [30, 31]	↓ OCS bursts/SABA (proxy); limited direct data [30, 31]	↓ OCS courses (median 2 → 0) [30, 31]	↓ NPS versus placebo (POLYP 1/2) [30, 31]	↑ tolerance (62.5% tolerated 930 mg vs. 0% placebo) [30, 31]
Dupilumab (anti‐IL‐4Rα)	↑ ∼+12.6% predicted at 4 wks (AERD) [20]	↓ ∼34 points at 3 mo (AERD) [30, 31]	↑; 73% normal/near‐normal smell at 1–3 mo [30, 31]	↓ in real‐world AERD [30, 31]	↓ OCS use (median 2 → 0/yr in cohort) [30, 31]	↓ versus placebo (SINUS‐24/52) [30, 31]	↑ threshold in case series [30, 31]
Mepolizumab (anti‐IL‐5)	Trend +5–6% predicted (AERD) [3, 36]	↓ ∼17.7 points (AERD) [3, 36]	↑ smell/↓ anosmia severity (clinical) [3, 36]	↓ annual rate (AERD cohort) [3, 36]	Allowed taper; fewer on maintenance OCS [3, 36]	↓ NPS; ↓ surgery risk 57% (SYNAPSE; includes AERD) [3, 36]	NR/No data identified
Reslizumab (anti‐IL‐5)	↔/↑ (limited AERD‐specific data) [3, 36]	No significant change in AERD cohort [3, 36]	NR	NR	NR	NR	NR
Benralizumab (anti‐IL‐5Rα)	↑ lung function; AERD‐specific limited [3, 36]	↓/mixed; CRSwNP RCT positive, practice variable [3, 36]	Mixed/limited AERD data [3, 36]	↓ in severe eosinophilic asthma (ANDHI) [3, 36]	↓ maintenance OCS in responders (limited AERD data) [3, 36]	↓ NPS versus placebo (CRSwNP RCT) [3, 36]	NR
Tezepelumab (anti‐TSLP)	↑ (∼+0.23 L overall; consistent in AERD subgroup) [38, 40]	Sinonasal symptom benefit in CRSwNP (details vary) [76, 84]	↑ smell in AERD case report [76, 84]	↓ ∼83% in AERD subgroup (NAVIGATOR) [76, 84]	↓ OCS requirements (program data) [76, 84]	↓ NPS and congestion versus placebo (CRSwNP RCT) [76, 84]	NR

*Note:* Arrows indicate direction of effect (↑ improvement; ↓ reduction). NR = not reported.

Abbreviations: AERD, aspirin‐exacerbated respiratory disease; CRSwNP, chronic rhinosinusitis with nasal polyps; FEV_1_, forced expiratory volume in one second; NPS, nasal polyp score; NR, not reported; OCS, oral corticosteroids; RCT, randomized controlled trial; SABA, short‐acting beta agonist; SNOT‐22, 22‐item Sino‐Nasal Outcome Test; UPSIT, University of Pennsylvania Smell Identification Test.

Observational cohorts and real‐world studies were included with randomized controlled trials (RCTs) due to the lack of robust data from RCTs. When comparing the RCT data to the observational cohorts and real‐world studies, findings from observational cohorts were largely consistent with those reported in RCTs. Both study designs demonstrated clinically meaningful improvements in sinonasal symptoms, asthma control, and quality‐of‐life measures following biologic therapy. However, real‐world studies often included patients with more severe or refractory disease, longer follow‐up durations, and greater treatment heterogeneity, providing valuable information regarding treatment persistence, long‐term effectiveness, and safety that may not be fully captured in RCTs. Conversely, RCTs provided higher internal validity through standardized outcome assessment and placebo‐controlled comparisons but generally enrolled more selected patient populations (Table 3).

**TABLE 3:** Randomized controlled trials of efficacy of biologics in AERD.

Study	Phase	Population	Intervention	Comparator	Primary outcome	Key findings
Omalizumab for aspirin hypersensitivity and leukotriene overproduction in Aspirin‐exacerbated Respiratory disease. A randomized controlled trial [17]	Not explicitly stated (likely phase II)	*n* = 16 adults with AERD (diagnosed by systemic aspirin challenge); aspirin therapy after desensitization (ATAD) on drug versus placebo	Omalizumab	Placebo	Evaluating the difference in area under the curve of logarithm LTE_4_ versus time graph in patients in the intent to treat population during oral aspirin challenge	Increased aspirin tolerance (62.5% tolerated 930 mg cumulative on omalizumab vs. 0% placebo); decreased urinary LTE_4_ during challenge; fewer respiratory symptoms.
Efficacy and safety of omalizumab in nasal polyposis: 2 randomized Phase 3 trials [30]	Phase III	Adults with CRSwNP separated into POLYP 1 (*n* = 138) and POLYP 2 (*n* = 127) (AERD subgroup *n* = 72/265, 27.6%); 2 year outcomes in comparison to placebo	Omalizumab (with intranasal mometasone)	Placebo (with intranasal mometasone)	Change in NPS and NCS from baseline to week 24. Secondary endpoint includes change from baseline to week 24 in SNOT‐22 score.	Decreased NPS and daily NCS; improved SNOT‐22 and UPSIT versus placebo; supports sinonasal benefit including in AERD subgroup.
Defining the efficacy of omalizumab in nasal polyposis: A POLYP 1 and POLYP 2 subgroup analysis [31]	Phase III	CRSwNP trial participants; subgroups including AERD *n* = 72/265 (27.5%); 2 year outcomes in comparison to placebo	Omalizumab (with background mometasone)	Placebo (with background mometasone)	Change in NPS and NCS from baseline to week 24. Secondary endpoint includes change from baseline to week 24 in SNOT‐22, TNSS, and UPSIT	Confirmed efficacy on NPS, NCS, SNOT‐22, UPSIT in relevant subgroups including AERD.
Efficacy and safety of dupilumab in patients with severe chronic rhinosinusitis with nasal polyps (LIBERTY NP SINUS‐24 and LIBERTY NP SINUS‐52): results from two multicentre, randomised, double‐blind, placebo‐controlled, parallel‐group Phase 3 trials [32]	Phase III	Adults with severe CRSwNP; included AERD patients, *n* = 724, with AERD enrollment *n* = 204 (28%)	Dupilumab	Placebo	Change in NPS, NCS, sinus Lund‐Mackay CT scores from baseline to week 24 in the intent to treat population	Reduced NPS and sinus opacification; improved smell and symptoms; consistent benefits across subgroups (supports AERD efficacy).Dupilumab was considered well tolerated
Mepolizumab for chronic rhinosinusitis with nasal polyps (SYNAPSE): a randomised, double‐blind, placebo‐controlled, Phase 3 trial [37]	Phase III	CRSwNP adults, *n* = 407; ∼27% AERD (∼108/407)	Mepolizumab (with background mometasone)	Placebo (with background mometasone)	Change in endoscopic NPS from baseline to week 52, along with mean nasal obstruction VAS score during weeks 49–52 in the intent to treat population	Reduced endoscopic NPS; 57% lower risk of sinus surgery versus placebo
Efficacy of tezepelumab in patients with severe, uncontrolled asthma across multiple clinically relevant subgroups in the NAVIGATOR study [38]	Phase III	Severe asthma, *n* = 1061; AERD subgroup analyzed	Tezepelumab	Placebo	Evaluation of annualized asthma exacerbation rate (AAER) over 52 weeks across clinically relevant subgroups (including AERD)	83% reduction in annualized exacerbation rate in AERD subgroup versus placebo; benefits across eosinophil strata.
Efficacy of tezepelumab in severe, uncontrolled asthma: Pooled analysis of the PATHWAY and NAVIGATOR clinical trials [40]	Phase 2b, and phase III	Severe asthma, *n* = 1334; with AERD subgroup	Tezepelumab	Placebo	Evaluation of annualized asthma exacerbation rate (AAER) over 52 weeks across pooled subgroups. Secondary endpoints include AAER associated with hospitalization or ED visit, and change prebronchodilator FEV_1_, ACQ‐6, AQLQ(S)+12 from baseline over 52 weeks	Improved FEV1 by ∼0.23 L more than placebo in the overall asthma population, and this improvement was consistent in those with AERD. Better asthma control scores and quality of life, with no specific data.

Abbreviations: AAER, asthma exacerbation rate; AERD, aspirin‐exacerbated respiratory disease; AQLQ(S), Standardized Asthma Quality of Life Questionnaire; ATAD, aspirin therapy after desensitization; CRSwNP, chronic rhinosinusitis with nasal polyps; ED, emergency department; FEV_1_, forced expiratory volume in one second; LMS, Lund–Mackay score; LTE_4_, leukotriene E_4_; NCS, nasal congestion score; NPS, nasal polyp score; SNOT‐22, 22‐item Sino‐Nasal Outcome Test; TNSS, Total Nasal Symptom Score; UPSIT, University of Pennsylvania Smell Identification Test.

Although no validated treat‐to‐target strategy currently exists for AERD, management should emphasize regular reassessment of both upper and lower airway disease following biologic initiation. Treatment targets may include improvement in asthma control, reduction in exacerbations, decreased systemic corticosteroid use, improvement in SNOT‐22 and nasal polyp score, restoration of olfaction, reduced need for sinus surgery, and improved quality of life. Biomarkers including blood eosinophils, FeNO, and urinary LTE_4_ may provide complementary information regarding inflammatory activity but should be interpreted alongside clinical response. Patients with inadequate response after an appropriate therapeutic trial should undergo reassessment for adherence, comorbidities, and consideration of switching to an alternative biologic targeting a different inflammatory pathway.

### 5.1 Aspirin Therapy and Biologics

Some AERD patients receive ATAD plus a biologic, and in fact, biologics may stabilize airway inflammation and reduce the severity of desensitization reactions, with limited data suggesting improved tolerability when ATAD is performed on agents such as dupilumab or omalizumab [8, 17, 33]. In a retrospective AERD cohort of 70 patients, combination therapy with ATAD plus a biologic achieved superior multidomain outcomes compared with ATAD monotherapy, including greater improvements ACT (+5.0 vs. + 2.0), SNOT‐22 (−28 vs. −14), and OCS reduction (−76% vs. −38%) [43]. Patients receiving both therapies likely represent a more severe disease subset, yet have not demonstrated increased adverse events and often achieve improved overall disease control [4]. However, contemporary real‐world data indicate that ATAD alone may have limited long term use, with higher discontinuation rates of around 40% on ATAD, and around 20% on biologic therapies and approximately 25% of patients transition from ATAD to biologics because of inadequate symptom control or intolerance [42].

A key question for the future is how ATAD fits into biologic use. While ATAD was historically a highly effective mainstay for AERD management, biologics now allow some patients achieve control without ATAD [42, 43]. In current practice, ATAD is increasingly reserved for patients with residual sinonasal disease despite biologic treatment, and there may be a synergistic benefit when biologics are initiated before desensitization [17]. Ongoing studies will be required to define optimal sequencing and determine whether biologics, ATAD, or combination therapy should be prioritized as first‐line treatment in AERD [26, 84].

### 5.2 Long‐Term Outcomes and Disease Modification

Another future consideration is whether biologics can modify the course of AERD long‐term. At present, biologics are considered chronic therapies that provide symptom control rather than remission, and there is no evidence that they alter the natural history of AERD [26]. Whether early or sustained biologic intervention can prevent polyp recurrence or allow treatment discontinuation warrants investigation through longitudinal studies and registries.

### 5.3 Cost and Access

Cost and access remain important considerations, as biologics are expensive and insurance approval may be challenging, particularly when used off‐label for AERD. Future health‐economic studies may demonstrate that prevention of surgeries and hospitalizations with biologics is a cost‐effective measure in AERD, as it has already been shown in severe asthma [85]. This could expand access and affordability.

### 5.4 Future Directions

Finally, prospective comparative trials and real‐world registries are needed to refine biologic selection in AERD [26]. Head‐to‐head studies comparing IL‐4/IL‐13 blockade with IL‐5 or TSLP‐targeted therapies, as well as evaluation of emerging agents, such as depemokimab, verekitug, lunsekimig, lebrikizumab, itepekimab, etokimab and stapokibart, will be critical to establishing evidence‐based treatment algorithms. Additionally, a formal systematic review is needed to compare the efficacy of biologics in patients with AERD to assess the quality of evidence from RCT. Until such data are available, personalized, phenotype‐driven biologic selection remains central to optimizing outcomes in AERD.

## 6 Conclusion

Available evidence supports the use of biologic therapies as effective treatments for AERD, with meaningful improvements across asthma control, sinonasal disease, and quality of life [26]. Among currently available agents, dupilumab demonstrates the most consistent multidomain efficacy in observational and comparative studies, while anti–IL‐5 and anti–IL‐5Rα therapies provide benefit primarily in eosinophil‐driven disease [3, 20, 41]. Omalizumab remains an important option for selected atopic AERD phenotypes, particularly in patients with concomitant allergic asthma [17]. Tezepelumab represents a promising emerging therapy with broad efficacy across inflammatory profiles, although direct comparative data in AERD are not yet available [19]. Future prospective studies should evaluate biomarker‐guided biologic selection and treat‐to‐target strategies incorporating clinical phenotype, inflammatory biomarkers, and patient‐reported outcomes to optimize personalized management of AERD.

The landscape of AERD treatment is rapidly evolving and advancing. Biologics have opened new frontiers, turning a once intractable disease into one that can be effectively managed in most patients. Prospective head‐to‐head trials and real‐world registries are needed to define optimal sequencing, identify predictors of response, combining therapies strategically to further improve outcomes, and clarify the role of newer agents. The next decade should bring clearer answers on how to optimally harness biologics to achieve disease remission and an improved quality of life for AERD patients.

## Author Contributions

Conceptualization: J.K.P., J.K.H., K.K.L., methodology: J.K.P., A.J.P., data curation: J.K.P., A.J.P., investigation: J.K.P., J.K.H., K.K.L., validation: A.J.P., formal analysis: J.K.P., A.J.P., supervision: J.K.H., K.K.L., writing original draft: J.K.P., writing – reviewing and editing: J.K.P., A.J.P., J.K.H., K.K.L.

## Funding

The authors have nothing to report.

## Ethics Statement

There were no study participants to obtain informed consent from. The study was granted exempt by the appropriate institutional and/or national research ethics committee, Eastern Virginia Medial School Institutional Review Board, and certify that the study was performed in accordance with the ethical standards as laid down in the 1964 Declaration of Helsinki and its later amendments or comparable ethical standards.

## Conflicts of Interest

Joseph K Han has received consultancy fees from Sanofi Genzyme, Regeneron, Genetech, Novartis, GSK and AstraZeneca; Kent K Lam served as a consultant for GSK and AstraZeneca. No other authors have relevant affiliations or financial involvement with any organization or entity with a financial interest in or financial conflict with the subject matter or materials discussed in the manuscript. This includes employment, consultancies, honoraria, stock ownership or options, expert testimony, grants or patents received or pending, or royalties.

## Data Availability

Data sharing not applicable to this article as no datasets were generated or analyzed during the current study.
